# Single-Walled Carbon-Nanotubes-Based Organic Memory Structures

**DOI:** 10.3390/molecules21091166

**Published:** 2016-09-02

**Authors:** Sundes Fakher, Razan Nejm, Ahmad Ayesh, Amal AL-Ghaferi, Dagou Zeze, Mohammed Mabrook

**Affiliations:** 1 School of Electronic Engineering, Bangor University, Dean Street, Bangor LL57 1UT, UK; s.j.fakher@bangor.ac.uk or fsundes@gmail.com; 2 Department of Electrical Engineering, United Arab Emirates University, Al Ain, UAE; r.radwan@uaeu.ac.ae; 3 Department of Mathematics, Statistics and Physics, Qatar University, Doha, Qatar; ayesh@qu.edu.qa; 4 Masdar Institute, Abu Dhabi, UAE; aalghaferi@masdar.ac.ae; 5 School of Engineering and Computing Sciences, Durham University, Durham DH1 3LE, UK; d.a.zeze@durham.ac.uk

**Keywords:** organic memory devices, single-walled carbon-nanotubes, pentacene, charge transfer

## Abstract

The electrical behaviour of organic memory structures, based on single-walled carbon-nanotubes (SWCNTs), metal–insulator–semiconductor (MIS) and thin film transistor (TFT) structures, using poly(methyl methacrylate) (PMMA) as the gate dielectric, are reported. The drain and source electrodes were fabricated by evaporating 50 nm gold, and the gate electrode was made from 50 nm-evaporated aluminium on a clean glass substrate. Thin films of SWCNTs, embedded within the insulating layer, were used as the floating gate. SWCNTs-based memory devices exhibited clear hysteresis in their electrical characteristics (capacitance–voltage (*C*–*V*) for MIS structures, as well as output and transfer characteristics for transistors). Both structures were shown to produce reliable and large memory windows by virtue of high capacity and reduced charge leakage. The hysteresis in the output and transfer characteristics, the shifts in the threshold voltage of the transfer characteristics, and the flat-band voltage shift in the MIS structures were attributed to the charging and discharging of the SWCNTs floating gate. Under an appropriate gate bias (1 s pulses), the floating gate is charged and discharged, resulting in significant threshold voltage shifts. Pulses as low as 1 V resulted in clear write and erase states.

## 1. Introduction

Due to the increased use of portable electronic devices, research has been devoted to developing new materials and structures for several applications in plastic technology. Applications such as organic solar cells [1,2,3], organic thin film transistors (OTFTs) [4,5,6], light emitting displays [7], radio frequency identification (RFID) tags [8] and sensors [9] have been evaluated in the last few years. Such devices attracted electronic engineering, physics, and chemistry researchers due to their flexibility, low cost, and low temperature processing.

Organic memory devices play a major role in plastic and flexible electronic applications [10], which lead to extensive research in this domain. Organic memory devices, based on charge storage in bistable switching [11,12,13], metal-insulator-semiconductor structures (MIS) [14,15,16,17], and organic thin film memory transistors (OTFMTs) [18,19,20,21] have been reported in the last 20 years. One of the well-known memory structures is the floating gate-based memory device; where typically a thin film of a floating gate serves as a charge storage node in a transistor or MIS structure. It is reported that stable memory behaviour can be achieved by charging nanoparticles or nanowires, which are integrated into the insulating layer of the MIS structures or thin film transistors [14,19]. When a program voltage is applied between the gate and the source contact, electronic charges can be transferred onto the floating gate by quantum tunneling or thermal emission, charging the floating gate and changing the transistor’s threshold voltage. A threshold voltage shift can be detected by measuring the drain current at a certain gate-source voltage. As the dielectric layer isolates the floating gate, charges stored on the floating gate remain there without the need for any applied voltage (nonvolatile memory). To erase the memory, a voltage of opposite polarity should be applied to discharge the floating gate [14].

A thin film of carbon-nanotubes (CNTs) is one of the most promising candidates for creating the ubiquitous memory devices for the next generation of nanoelectronics. Due to the high thermal stability, tuneable bandgap, high aspect ratio, nearly zero surface states, chemical inertness [22], as well as the excellent electrical properties and mechanical strength [23]; CNTs is well suited for memory applications where they may act as a charge storage element in floating gate memory structures. In principle, the merging of the essential memory functionality and the exceptional electrical properties of the nanotubes may open a new route to nanoscale memory devices with ultrahigh integration density. On the other hand, many research groups worldwide have thoroughly investigated carbon nanotube field-effect transistors (CNTFETs) [24] as a promising replacement for silicon-based field effect transistors. Nevertheless, in this kind of field-effect transistors, several issues have to be resolved prior to large-scale integration. One of these issues is the hysteresis in the transfer characteristics of the CNTFETs, which seems to be an intrinsic property [25]. However, in order to be used as a FET-substitute in logic circuits, which is of course not the case in the presence of a pronounced hysteresis effect; it should have very defined parameters such as a threshold voltage. Otherwise, the hysteresis becomes a big advantage when using the CNTFETs as memory devices instead. In recent progress, we have reported floating gate memory devices based on the MIS and transistor structure, using a thin film of Au layer [8,26] where there is a clear hysteresis in their electrical characteristics for the transistor and also for the capacitance–voltage (*C–V*) characteristics of MIS structures. On the other hand, there is little or no hysteresis for the control devices of these structures. Furthermore, in our previous work [14,19], we have reported on MIS and transistor structures in which a monolayer of gold nanoparticles was incorporated into insulating films.

Here, we report on the fabrication and characteristics of organic MIS and field effect transistor-based floating gates memory structure. We employ the performance of non-volatile memory effects of a single-walled carbon-nanotubes (SWCNTs) based structure. The polymethylmethacrylate (PMMA) was used as the dielectric layer, and pentacene as the active layer in these devices. Layer-by-Layer assembled composites were used for deposition of SWCNTs as the charge storage layer. Achieving a large memory window, which gives an indication of the potential for high-density charge storage, was the major feature of this work.

## 2. Results and Discussion

### 2.1. C–V Characteristics for MIS Memory Devices

**Figure 1:**
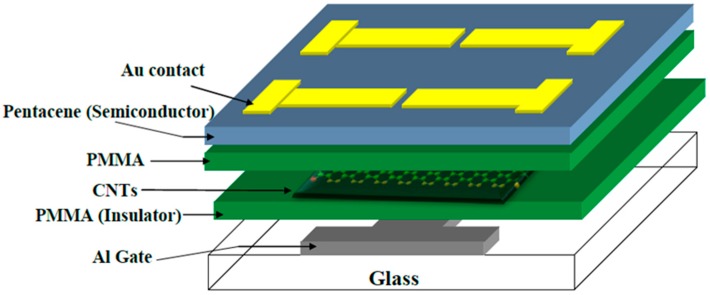
Schematic diagram of the single-walled carbon-nanotubes (SWCNTs)-based organic thin film memory transistors (OTFMT) with SWCNTs floating gate.

**Figure 2:**
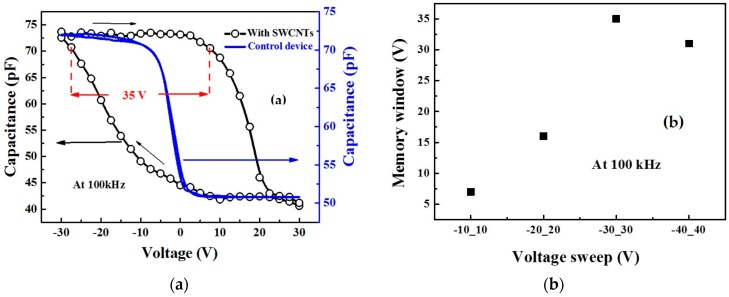
(**a**) *C*–*V* characteristics at 100 kHz for the SWCNTs-based metal–insulator–semiconductor (MIS) memory (open circle and line) and control (blue solid line) devices and (**b**) the memory window (flat–band voltage shift) versus voltage sweep range.

Figure 1 illustrates the general configuration of the organic MIS memory structure and organic thin film memory transistor (OTFMT) based on SWCNTs as the floating gate. The *C–V* characteristics for SWCNTs-based MIS memory structure, and the control device (without SWCNTs), are shown in Figure 2a. All the measurements reported in this work were performed at 100 kHz and at a voltage scan rate of 2 Vs^−1^. In each measurement, the double scan started from a negative gate voltage, swept towards the inversion region, and then back to negative voltages. The *C–V* curve for the control device (blue solid line in Figure 2a), with a reference structure of Al/PMMA/pentacene/Au, reveals the typical characteristics of an MIS structure based on a p-type semiconductor, with a flat–band voltage of about −8 V and full semiconductor depletion at about 1 V. Negligible hysteresis is evident at the double voltage sweep rate used for the control device. The measured value of the accumulation capacitance for the reference device was 72 pF. This was consistent with the insulator thickness ~330 nm and the device area 9 × 10^−3^ cm^−2^. On the other hand, the additional layers of SWCNTs within the insulating stack to form an Al/PMMA/SWCNTs/PMMMA/pentacene/Au structure, produced a very noticeable change in the flat–band voltage. Furthermore, on reversing the direction of the voltage scan, significant hysteresis and a shift in the flat–band voltage were observed in the *C*–*V* curve with a large memory window ΔV ~ 35 V, which is indicative of the charge storage in the SWCNT layers. As shown in Figure 2a, if a certain voltage (less than −30 V) is applied, the device will be in the high accumulation capacitance and thus perform the write operation of the ON state. On the other hand, if a voltage of higher than 25 V is applied (deep depletion region) the erase operation will perform and the device turns to its OFF state. Applying a voltage between −30 V and 25 V represents the reading voltage range at which the capacitance value indicates if the device is in the ON or OFF state without changing it.

The memory devices exhibited a larger shift in the flat-band voltage as the voltage sweep range increased, as shown in Figure 2b. The scan rate was kept at a constant value of 2 Vs^−1^ to ensure measurement optimization. The increase in the memory window, with increasing sweep voltage, is expected as more charges are introduced and stored in the same period. However, when the voltage sweep reached ±40 V, the memory window slightly decreased due to the polarization effect from the SWCNTs stack. A similar increase in the memory window, with an increasing voltage sweep, was reported by several groups [27,28].

The clockwise hysteresis after sweeping the accumulation was associated with electrons charging of floating gate or polarization of the insulator. Furthermore, it is clearly seen in Figure 2 that the hysteresis centred on approximately 0 V, which indicates that the devices may operate at lower voltages. The amount of charge stored in the carbon nanotubes Q can be estimated depending on the C_i_, which gives a value of 8.2 × 10^−9^ F·cm^−2^, and the ΔV_FB_ was 35 V; the maximum number of charge carriers stored is approximately 1.7 × 10^12^ cm^−2^. These results are consistent with our recent studies using different memory stacks [14]. The clockwise direction of the hysteresis, with a shift of the flat–band voltage to a less negative voltage, indicates that electrons originating from the Al gate become trapped on the floating gate. In this case, in accumulation, electrons injected from the Al electrode to the SWCNTs layer later become negatively charged. The opposite effect occurs in inversion, and electrons are transferred to the Al electrode from the SWCNTs floating gate.

### 2.2. I–V Characteristics for TFMT Devices

**Figure 3:**
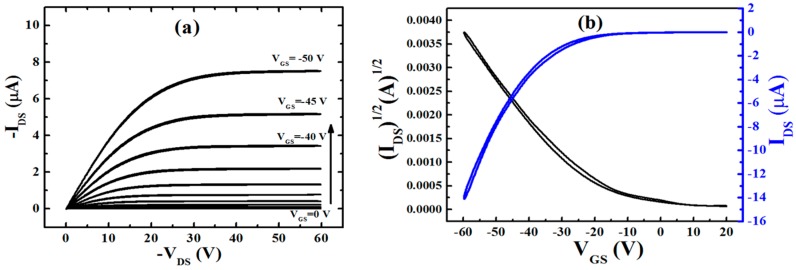
(**a**) Output characteristics for the organic thin film transistors (OTFT) control device; (**b**) Transfer characteristics for OTFT.

The output characteristics of the fabricated organic thin film transistor (OTFT) control devices (without CNTs floating gate) with the structure of Al/PMMA/pentacene/Au are shown in Figure 3. The only difference compared to the memory device is the absence of SWCNTs trapping layers in the gate dielectric layer. Otherwise, all of the device structures and the processing conditions are the same. Figure 3 shows the typical output characteristics (the dependence of drain–source current I_DS_ on the drain–source voltage V_DS_) of the p-channel OTFT devices, with respect to the gate bias from 0 to −50 V with steps of 5 V. The transfer characteristic (the dependence of I_DS_ on the gate–source voltage V_GS_) of the organic transistor is shown in Figure 3b for V_DS_ = −25 V. A negligible hysteresis and gate leakage current are evident in both characteristics at the voltage sweep rates that are used for the control device.

**Figure 4:**
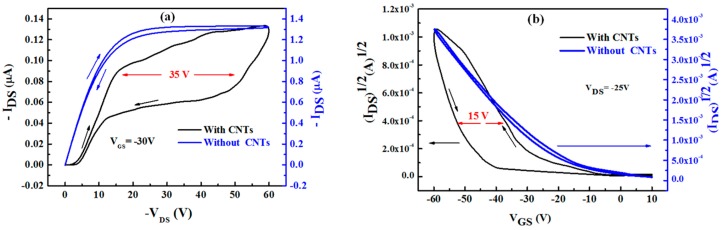
(**a**) The output and (**b**) transfer characteristics of the OTFMT device with and without the SWCNTs floating gate.

The transistor behaviour of the memory devices was investigated by measuring the output and transfer characteristics of the OTFMT at room temperature. Figure 4a shows the output characteristic of SWCNTs-based OTFMT and the OTFT control device, which is associated with memory structure. Both forward and reverse scans are shown in each measurement at a voltage scan rate of 2 Vs^−1^ and with a V_GS_ value of −30 V. The output characteristics of the memory and control devices exhibited good linear behaviour at low V_DS_ values, as well as a good saturation region at high V_DS_. It is also evident from Figure 4a that the memory transistor shows negligible gate leakage current, indicating good transistor behaviour. Figure 4b shows the transfer characteristics of the memory device (initial curve) as well as the control device; the V_DS_ value was set at −25 V. The channel length was L = 147 μm, and the channel width was W = 1000 μm for this memory device.

The addition of the SWCNTs floating gate produces a clear hysteresis in both the output and the transfer characteristics of the transistor, as shown in Figure 4. The hysteresis is the result of the charging and discharging of the SWCNTs floating gate with the appropriate applied voltages. When a high enough negative bias is applied to the gate electrode, holes are injected from the semiconducting layer into the SWCNTs floating gate (through the top insulating layer), charging up the SWCNTs floating gate and programming the memory device. On the other hand, when a high enough positive voltage is applied to the gate electrode, holes are ejected from the floating gate through the pentacene layer (erase process). Large memory windows were clearly evident in Figure 4; a memory window of ΔV = 35 V was observed in the output characteristics and a memory window of ΔV = 15 V was observed in the transfer characteristics. Such a large memory window for SWCNTs-TFMT devices depends significantly on dV_G_/dt, which is the sweeping rate of the gate voltage due to the charge storage effect [29]. A clockwise hysteresis loop is observed for the output characteristic, whilst a counter-clockwise hysteresis loop is observed for the transfer characteristic when the gate voltage sweeps from positive to negative voltages. Such hysteresis loops are in satisfactory agreement with our recent studies for a gold thin layer [26] and for gold nanoparticles [30]; these results are consistent with other reports [31] for CNT based TFMT devices.

The field-effect mobility μ of the devices can be estimated from;

(1) $$I_{\mathrm{DS}}=\frac{\mu WC_{i}}{2L}{\left(V_{\mathrm{GS}}-V_{T}\right)}^{2}$$

where C_i_ is the insulator capacitance per unit area and V_T_ is the threshold voltage. The threshold voltage represents the value of the V_GS_ at which the transistor is turned on and can be determined from the intercept of the plot of (I_DS_)^1/2^ versus V_GS_, as shown in Figure 4b. The calculated value of the field-effect mobility μ for the control device was 0.212 cm^2^·V^−1^·s^−1^, with a threshold voltage of −18 V and an on/off current ratio of 2.9 × 10^3^. Besides the large memory window exhibited in SWCNTs-OTFMT devices, a relatively good field effect mobility; µ = 0.319 cm^2^·V^−1^·s^−1^ has been observed. The threshold voltages were estimated at about −22 and −45 V for forward and reverse directions respectively, and the on/off current ratio was 0.23 × 10^3^. The amount of charges stored in the carbon nanotubes (Q) can be estimated depending on the C_i_ (10.53 × 10^−9^ F·cm^−2^) and ΔV_T_ (35 V). The number of charge carriers stored was calculated to be in the range of 1.7 × 10^12^ cm^−2^.

**Figure 5:**
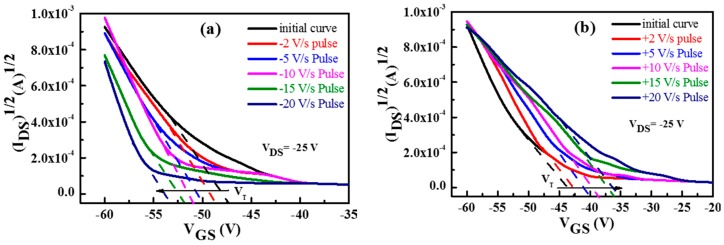
The effect of (**a**) negative and (**b**) positive pulses on transfer characteristics for SWCNT-based OTFMT.

**Figure 6:**
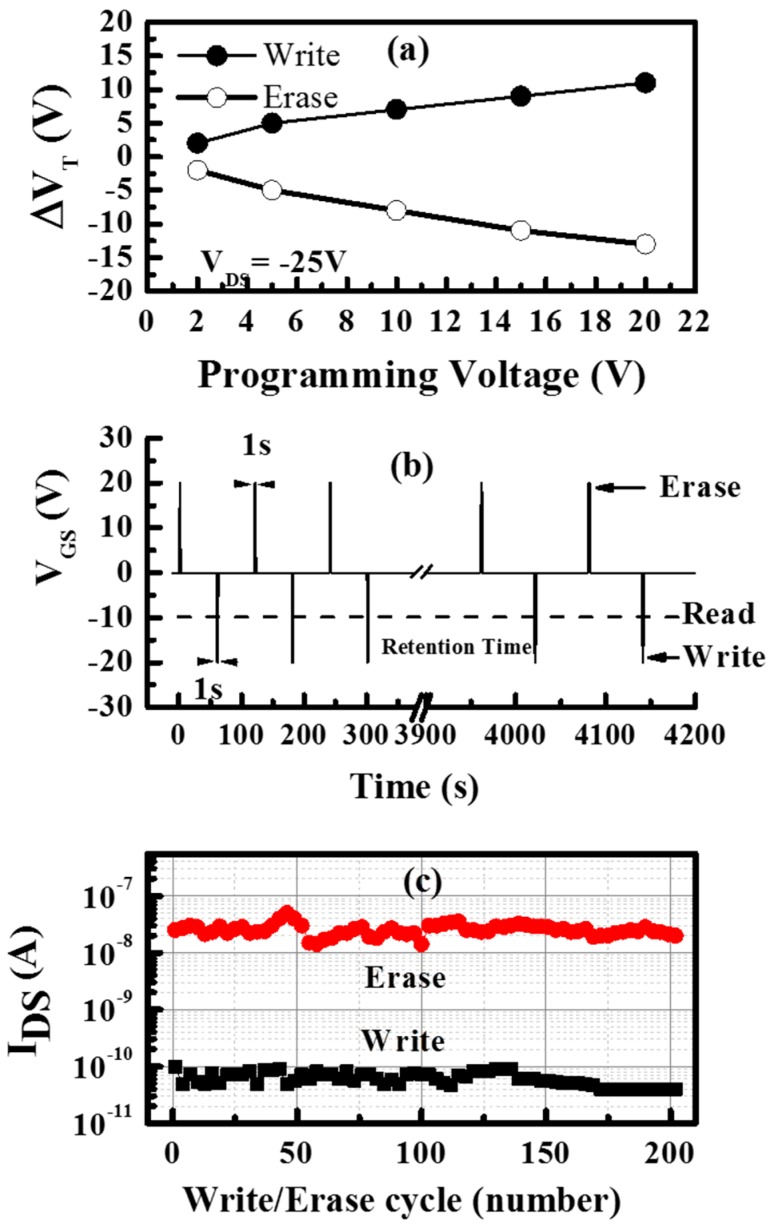
(**a**) Programming characteristics; (**b**) pulses sequence and (**c**) retention current for the SWCNT-based OTFMT.

The memory operation was again characterised by measuring the threshold voltage shift after charging the floating gate, as successive positive and negative voltage pulses were applied to the gate electrode with V_DS_ maintained at 0 V. The magnitude of the voltage pulse was increased for each step but the pulse duration was kept at 1 s. The transfer characteristic of the device was measured after each application of the voltage pulse to calculate the shift in the threshold voltage compared to the unstressed device. Figure 5 shows the effect of negative and positive pulses applied to the gate electrode of the SWCNT-based OTFMT. A clear shift to higher negative threshold voltages is observed for the application of negative pulses (write state) as shown in Figure 5a, whereas, positive shifts of threshold voltages are observed due to the application of positive pulses (erase) as shown in Figure 5b. Figure 6a shows the programming pulses of SWCNT-based OTFMTs, where the threshold voltage shift is a result of the applied negative and positive pulses. The write state happens when negative pulses (1 s pulses here) are applied to the gate electrode, leading to the threshold voltage shifting to more negative values compared to the unstressed device, while the application of a positive pulse to the gate electrode leads the transfer characteristic to shift rigidly toward positive gate voltages, the erase state. These behaviours are evident in Figure 6a as the shift in threshold voltage increases with an increase in the applied voltage pulses. Clear memory behaviour, in terms of writing and erasing for voltage pulses as low as 2 V, are shown in Figure 6a. It is also important to report that the mobility of memory transistors did not vary due to the applied write or erase pulses as mobilities of 0.3 to 0.33 cm^2^·V^−1^·s^−1^ were estimated for all conditions.

The nonvolatile behaviour of the OTFMTs—for the purpose of measuring the endurance properties—was investigated by monitoring the drain-source current after the application of voltage pulses for write and erase states. The write/erase operations were repeated with continuous application of bias pulses of ±20 V for 1 s. After a certain number of write/erase cycles, the reading process was carried out to confirm the change in the drain current. Figure 6b shows the test pulse sequence for endurance measurements of the SWCNT-based TFMTs device at room temperature. As shown in Figure 6b, there are three write/erase cycles in the first period with an initial decay of 60 s for each cycle, and then the drain current is measured by an applied reading bias of −10 V. After a retention time of one hour, the write/erase operations were repeated (two cycles, as shown in Figure 6b) and were followed with a reading process. This process was repeated for over 200 cycles and the I_DS_ value of the memory transistor was measured accordingly. It is observed that the current remains almost constant in both write and erase states as shown in Figure 6c. The average currents recorded for the write and erase states are 7.12 × 10^−11^ A and 2.5 × 10^−8^ A, respectively.

**Figure 7:**
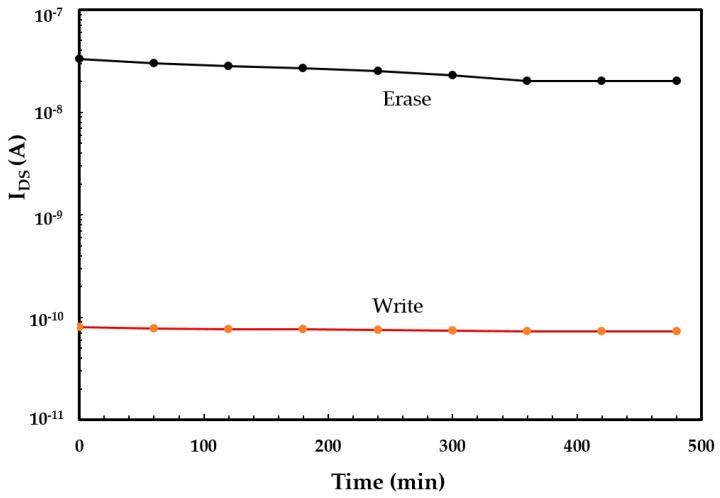
Charge retention capability as a function of retention time for the SWCNT-based OTFMT.

The nonvolatile behaviour of the OTFMT was also investigated by monitoring I_DS_ after the application of voltage pulses for write and erase states. I_DS_ was periodically measured at regular time intervals with a fixed reading voltage of 10 V. Figure 7 shows the data retention capability as a function of time for SWCNTs-based OTFMT in the write/erase states in an ambient condition at room temperature. In fact, the memory behaviour was retained for more than 12 months in the case of OTFMTs stored in a vacuum.

**Figure 8:**
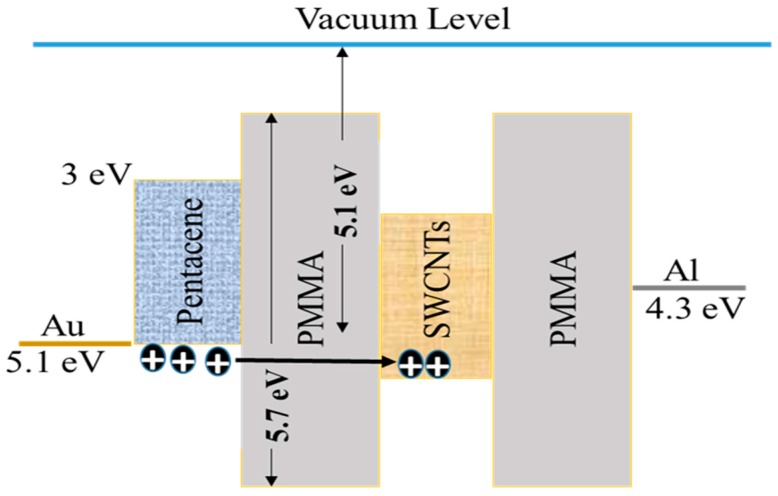
Energy band diagram of the SWCNT-based memory transistor.

Figure 8 represents the relative energy diagrams for the materials used in the fabrication of the device depicted in Figure 1, where the work functions for Al, Au, and SWCNTs are 4.3, 5.1 and 4.8 eV, respectively. The highest-occupied molecular orbital (HOMO) and the lowest-unoccupied molecular orbital (LUMO) levels of pentacene are −5 eV and −3 eV, respectively, while the energy band gap of PMMA is 5.7 eV with a work function of 5.1 eV [32]. In the charging process (writing) shown in Figure 8, where a negative bias is applied to the gate electrode, holes emitted from the highest-occupied molecular orbital (HOMO) level are injected through the PMMA and captured by the SWCNTs floating gate. The presence of holes in the insulating stack of the transistor leads to a higher negative voltage being required to activate the transistor (higher negative threshold voltage). Based on the experimental results and the energy band diagram in Figure 8, we believed that the transfer of holes from the pentacene to the SWCNTs floating gate occurs by tunnelling through the PMMA. The charge transfer may be supported by the localised defects present in the SWCNTs floating gate. Also, charge carriers can cross the PMMA energy barrier as the HOMO level of pentacene and the work function of SWCNTs are very close (as shown in Figure 8). This is characterised by a large negative shift in the threshold voltage of the transfer characteristics during the reverse sweep. In turn, erasing occurs when a positive bias is applied to the gate electrode.

Reproducible memory properties were observed, with OTFMTs stored in a vacuum, as devices were tested on a regular basis for 12 months. This indicates a potential for the proposed SWCNTs-based OTFMT as a low cost, non-volatile, memory device. The results augur well for the development of all-organic, flexible circuitry, for future plastic technology.

## 3. Materials and Methods

The TFTs memory devices, based on SWCNTs as a nanofloating gate of structure Al/PMMA/CNTs/PMMA/pentacene/Au, are depicted in Figure 1. The following materials were purchased from Sigma-Aldrich (Dorset, UK): PMMA (molecular weight 93,000) and pentacene. The device was fabricated by thermally evaporating a 50 nm thickness of an Al gate electrode through a shadow mask onto a clean glass substrate. A 300 nm thick insulating layer was formed by spin coating an anisole solution of PMMA on top of the gate electrode and curing at 120 °C for 1 h. The PMMA solution concentration and spin coating speed were 10% (wt) and 5000 rpm, respectively. Single walled carbon nanotubes were purchased from Carbon Nanotechnologies Inc. Before their integration into the memory devices, SWCNTs were purified until the metal content was below 5 wt %. In the purification process of the nanotubes, SWCNTs were subjected to a thermal oxidation for 90 min at 300 °C, followed by stirring in a concentrated HCl bath overnight, before finally rinsing the nanotubes with deionized water until the pH of the solution is equal to that of deionized water, and drying overnight at 120 °C. The SWCNTs were then subjected to a chemical cutting process using mild sonication in a mixture of H_2_SO_4_ and HNO_3_ (in the ratio 1:1) for 3 h at 120 °C. The SWCNTs were separated by centrifugation, washed several times with deionized water, and dried for 18 h. The cut SWCNTs were filtered using the 0.2 μm pore size polycarbonate membranes. At the end of the procedure SWCNTs of 200 nm or shorter were produced. The floating gate of SWCNTs was deposited using a Layer-by-Layer (LbL) technique; a deposition technique based on a charge reversal to build up bi-layer assemblies of oppositely charged molecules [33]. Briefly, the deposition began by functionalizing the substrate by seeds layers, which facilitates the adhesion of SWCNTs onto the substrate. This was performed by the alternate immersion of the substrate in aqueous Poly(ethyleneimine) (PEI) (Mw = 25,000) (cationic, pH = 8.5) and Poly(acrylicacid) (PAA) (Mw = 4,000,000) (anionic, pH = 6.5) solutions, for 15 min each. The substrate was then repeatedly immersed in PEI solution for 15 min, then SWCNTs solution (anionic, functionalized SWCNTs dispersed in Sodium dodecyl sulphate (SDS) solution) for 30 min. After each immersion, the substrate was rinsed with deionized water and dried with nitrogen. The final SWCNTs matrix consisted of three SWCNTs–PEI bilayers with a thickness of 10–20 nm. Prior to the deposition of the pentacene semiconducting layer, another 300 nm thin film of PMMA was deposited onto the floating gate. Pentacene was thermally evaporated at a pressure of 7.5 × 10^−7^ mbar, at a rate of 0.03 nm·s^−1^, through a shadow mask, to a thickness of 50 nm. Following deposition of the pentacene, the contact was defined by thermal evaporation of 50 nm of Au through a shadow mask. The channel width was W = 1000 μm and the channel length was L = 147 μm. The control device without SWCNTs was also fabricated. Double sweep capacitance voltage (*C*–*V*) measurements were carried out (using a LCR Bridge (HP4192) at 100 kHz and a typical 2 Vs^−1^ scan rate) to investigate the memory behaviour of the specimens produced. Double sweep current (*I*) versus voltage (*V*) characteristics of the transistors were recorded at room temperature (21 ± 2 °C) using a Keithley 4140B picoammeter.

## 4. Conclusions

Organic memory devices based on MIS and transistor structures using SWCNTs as charge storage are fabricated and characterised. We have demonstrated in this work that SWCNTs can be utilised as a reliable storage element in organic memory devices. PMMA was used as dielectric layers and pentacene was used as the active layer. Large memory windows (Δ*V*_th_ > 35 V), as well as high carrier mobility (µ = 0.319 cm^2^·V^−1^·s^−1^), are demonstrated. The floating gate may be charged and discharged, resulting in a clear shift in the threshold voltage of the transistors, and flat-band voltage of the MIS structure, by applying appropriate negative or positive voltages pulses. Data retention properties for SWCNTs memory devices were expected from the extrapolation of the measured data retention characteristics, which showed the stored information was maintained for a long time and the hysteresis may be used as the basis of a stable memory at room temperature. However, improvements in the quality of the memory devices are needed before these devices can be used in future plastic technology. In particular, the number of writing/erasing cycles needs to increase to more than 1000 cycles.
